# Brugada syndrome risk scores: what we've learned and what's next

**DOI:** 10.3389/fcvm.2025.1715146

**Published:** 2026-01-30

**Authors:** Pattara Rattanawong, Win-Kuang Shen

**Affiliations:** 1 Sport and Genetic Electrophysiology Clinic, Pali Momi Medical Center, Hawaii Pacific Health, Honolulu, HI, United States; 2 Department of Cardiovascular Medicine, Mayo Clinic, Phoenix, AZ, United States

**Keywords:** Brugada syndrome, risk stratification, score, sudden death, ventricular fibrillation

## Abstract

Brugada Syndrome (BrS) is a rare but clinically significant inherited arrhythmia disorder characterized by a type 1 ECG pattern and an increased risk of sudden cardiac death (SCD). Since its first description in 1992, BrS has been the subject of intensive investigation, yet risk stratification remains one of its greatest challenges. While survivors of cardiac arrest and patients with documented ventricular fibrillation (VF) are clear candidates for implantable cardioverter-defibrillators (ICDs), predicting risk in asymptomatic or intermediate-risk individuals is less straightforward. Over the past two decades, multiple risk scores have been developed—including the Sieira, Shanghai, BRUGADA-RISK, and PAT—each integrating combinations of clinical, ECG, electrophysiological study (EPS), and genetic data. Performance metrics vary, with C-statistics ranging from 0.70 to 0.82 in derivation cohorts, but external validation has often been limited. Importantly, current ESC and AHA/ACC guidelines only endorse syncope and EPS inducibility as validated predictors, reflecting the cautious stance of expert panels in the face of heterogeneous data. Nonetheless, the emergence of structured risk models has improved our ability to stratify intermediate-risk patients and stimulated further innovation. Looking ahead, opportunities lie in integrating artificial intelligence applied to raw ECG waveforms, wearable technology for dynamic monitoring, advanced cardiac imaging biomarkers, and polygenic risk scores. Multinational collaboration and federated learning will be essential to overcome statistical fragility and ensure global applicability. Ultimately, BrS risk scores should be considered decision-support tools that enrich but do not replace clinical judgment. Shared decision-making remains central, particularly in asymptomatic patients where ICD implantation is not a clear-cut choice.

## Introduction

Brugada Syndrome (BrS) was first described in 1992 by the Brugada brothers as a distinct arrhythmogenic entity characterized by coved ST-segment elevation in the right precordial leads and an association with sudden cardiac death (SCD) (1). Its worldwide prevalence ranges from 0.3 per 1,000 to 0.7 per 1,000, though rates are higher in Southeast Asia, 35.5 per 1,000 (2–4) where sudden unexplained nocturnal death syndrome is likely a manifestation of the same condition (5). BrS exhibits striking sex-related differences: although inherited in an autosomal dominant manner, men are more likely to manifest the ECG phenotype and experience arrhythmic events (6). Genetic studies have identified pathogenic variants in SCN5A, encoding the cardiac sodium channel, but penetrance is incomplete, and many BrS patients lack identifiable mutations (5). Clinically, BrS spans a wide spectrum. Some patients remain asymptomatic throughout life, while others present with syncope, ventricular arrhythmias, or aborted cardiac arrest. Symptomatic individuals, particularly survivors of ventricular fibrillation (VF) or SCD, are unequivocally considered high risk and managed with implantable cardioverter-defibrillator (ICD) implantation (7). However, the management of asymptomatic individual remains controversial (8–10). Their absolute event rate is relatively low, yet ICDs carry substantial long-term risks including inappropriate shocks, lead malfunction, infection, and psychological burden (10). Consequently, the central challenge in BrS management is accurate risk stratification. Although numerous predictors have been proposed, international guidelines currently endorse only two—arrhythmic syncope and electrophysiology study (EPS) inducibility—as validated markers sufficient to guide ICD implantation (7, 11). To improve precision, multiple multivariable scores have been developed, but none have been adopted into guidelines (12–15).

### Evolution of risk stratification

The earliest management approach to BrS was binary: patients with prior VF or SCD were deemed high risk and treated with ICDs, whereas asymptomatic individuals were observed (7). While simple, this approach was challenged by registry data showing that a substantial proportion of arrhythmic events occurred as the first manifestation (16). The FINGER registry (France, Italy, Netherlands, Germany) enrolled over 1,000 patients and demonstrated that spontaneous type 1 ECG, history of syncope, were independent predictors of arrhythmic events but not inducibility at EPS, family history of SCD, and *SCN5A* mutation (17). It also highlighted that low-risk classifications often failed, as some asymptomatic patients experienced sudden events. The PRELUDE registry, focusing specifically on asymptomatic individuals, confirmed the prognostic significance of syncope but revealed variability in predictive value of EPS depending on stimulation protocols. Fragmented QRS and early repolarization patterns emerged as potential markers, but reproducibility was limited and definitions inconsistent (17). The multinational SABRUS registry, which included both Asian and Western cohorts, demonstrated that the overall age of first arrhythmic event was similar across ethnicities, with no significant earlier onset in Asians. However, Asian patients were more likely to present with aborted cardiac arrest as their first event and had a higher proportion of male patients compared to Western cohorts. Additional differences included a lower prevalence of SCN5A mutations in Asians, highlighting important ethnic variability in BrS expression and outcomes (18, 19). Collectively, these registries shifted practice from symptom-based classification toward multivariable risk assessment, laying the groundwork for structured risk scores.

### Major risk scores

Over the past two decades, multiple risk scores have been proposed to improve prediction of arrhythmic outcomes in BrS (Table 1). Each score integrates clinical and electrocardiographic features, and in some cases EPS results and genetic data, to generate a structured assessment of risk. While these models represent progress beyond binary classification of “symptomatic vs. asymptomatic,” their derivation cohorts, methodologies, and validation results differ considerably.

**Table 1:** Comparison of risk predicting score in Brugada syndrome.

Model	Year	Derivation cohort	Core predictors (examples)	Endpoint	Pooled discrimination	Advantages	Limitations
Delise et al. algorithm	2011	Multicenter in Italy	Syncope, family history of SCD, positive EPS, spontaneous type 1	Appropriate shock and MAE	First MAE AUC ∼0.70; first/recurrent AUC ∼0.66	Simplicity; easy bedside variables	Not a score-based algorithm; derived from a small cohort; limited external validation
Sieira et al. score	2017	Single-center (Brussels)	Syncope, aborted SCD, spontaneous type 1, early SCD in first-degree relatives, sinus node dysfunction, inducible VA	First MAE	First MAE AUC ∼0.73; first/recurrent AUC ∼0.79	Clinically intuitive; easy bedside variables	Single-center derivation; external validation; underperforms when compared to BRUGADA-RISK and PAT
Shanghai score system	2018	Consensus diagnostic score; validated in Japanese registry	Spontaneous/fever/drug-induced type 1, arrhythmic or unexplained syncope, family history, SCN5A	Primarily designed for diagnosis	First event AUC ∼0.69; first/recurrent AUC ∼0.76	Widely adopted; structured for diagnosis; prognostic gradient observed	Optimized for diagnosis—not risk; lower discrimination for MAE
BRUGADA-RISK	2021	Multicenter	Arrhythmic syncope, spontaneous type 1, early repolarization, type 1 in peripheral leads	First VA or SCD	First MAE AUC ∼0.81; first/recurrent AUC ∼0.74	Designed for primary prevention; multicentre; relatively parsimonious	Variable external performance; recurrent MAE prediction weaker than PAT in pooled analyses
PAT (Predicting Arrhythmic evenT)	2023	Worldwide pooled analysis; internal & external validation	TpTe>100 ms, Syncope, VAs during drug challenge testing, prolonged PR, type 1 in peripheral leads, aVR sign, fQRS, and ER	First and recurrent MAE	First MAE AUC ∼0.79; first/recurrent AUC ∼0.84	Best for combined first/recurrent events; broad, multiparametric; reflects diverse cohorts	Variable external performance; first MAE prediction weaker than BRUGADA-RISK

BrS, Brugada syndrome; MAE, major arrhythmic events; VA, ventricular arrhythmia; VF, ventricular fibrillation; ER, early repolarization; fQRS, fragmented QRS; AUC, area under the ROC curve.

The Delise model, from an Italian cohort, combines spontaneous type 1 ECG, syncope, family history, and EPS inducibility, identifying highest risk when ≥2 factors are present (20). The Kawazoe score, derived in Japan, emphasizes noninvasive ECG markers such as fragmented QRS and Tpeak–Tend dispersion, showing good discrimination in its derivation cohort (21). The Okamura model incorporates spontaneous type 1 ECG, syncope, and programmed stimulation inducibility as key predictors of future events (22). These models reflect heterogeneous strategies—clinical, ECG, and EPS-based—but each has limited external validation. Overall, they provide complementary insights yet highlight the need for more broadly applicable risk tools.

The Sieira Score, derived from a large Spanish registry of nearly 1,000 patients followed for more than eight years, is one of the earliest validated risk models in BrS (14). It incorporates four variables: spontaneous type 1 ECG, history of syncope, presence of sinus node dysfunction, and inducibility at EPS. Weighted points are assigned to each factor, with cumulative scores stratifying patients into low-, intermediate-, and high-risk categories. In derivation, spontaneous type 1 ECG carried a hazard ratio (HR) of approximately 2.7 for arrhythmic events, history of syncope with HR of 2.7, sinus node dysfunction of 5.0, while inducibility at EPS carried an HR near 4.7. The overall discriminatory ability was modest, with a C-statistic area under curve (AUC) of around 0.73 (23). Strengths of the Sieira Score include its simplicity and clinical familiarity. However, the reliance on EPS—a controversial predictor with inconsistent reproducibility across centers—limits its universal applicability. Additionally, its development in a primarily European cohort raises questions regarding external generalizability, particularly in Asian populations where BrS expression differs.

The expert-consensus-driven Shanghai Score was originally designed as a diagnostic framework to standardize BrS definition and distinguish it from phenocopies (15). The score assigns points for spontaneous or drug-induced type 1 ECG, arrhythmic symptoms, family history of sudden death, and presence of a pathogenic genetic mutation. Although highly valuable for diagnosis, its prognostic application is less robust. When applied to risk prediction, the Shanghai Score demonstrates high sensitivity but low specificity (24). Several validation attempts show C-statistics AUC between 0.69 to 0.80 (12, 25), with limited ability to separate low- and intermediate-risk individuals. Thus, while the Shanghai Score is widely adopted in diagnostic practice, it is not recognized as a standalone prognostic tool. Nevertheless, it emphasizes the value of integrating genetic and family history data into broader risk frameworks.

The BRUGADA-RISK score represents a modern, single-cohort-driven approach (13). Developed from a multinational cohort exceeding 2,000 patients, it applies advanced statistical and machine-learning methods to derive individualized risk probabilities. Variables include spontaneous type 1 ECG, syncope, and selected ECG markers including type 1 ECG and early repolarization in peripheral leads. In validation cohorts, BRUGADA-RISK demonstrated C-statistics 0.81, slightly outperforming the Predicting Arrhythmic evenT (PAT) score (AUC 0.79) but significantly outperforming the Sieira Score (AUC 0.73) (23). It also generates continuous probability estimates rather than categorical cutoffs, aligning more closely with individualized medicine. However, its limitations include the need for complete datasets and computational infrastructure, which may limit use in routine clinical practice. Moreover, machine-learning models face challenges regarding interpretability—a barrier to clinician trust and adoption.

The PAT Score was designed to address limitations of earlier tools. Unlike most prior models, the PAT Score incorporates both first and recurrent arrhythmic events as outcomes (12). This is a key innovation, as patients with BrS remain at lifelong risk, and focusing solely on initial events underestimates overall arrhythmic burden. It was derived from a systematic review and meta-analysis of BrS risk factors from 67 studies (7,358 patients), then converted the significant pooled odds ratios into a weighted, linear risk score. The PAT Score achieved a C-statistic of about 0.95 in the truly unpublished external validation cohort, with particular strength in intermediate-risk patients—a historically difficult subgroup to stratify. The score incorporats readily available clinical symptoms and ECG findings (12). In the Japanese cohort, internal validation demonstrated lower discriminatory performance of the PAT score compared with the BRUGADA-RISK score for predicting first events (AUC 0.61 vs. 0.73), but higher discriminatory performance for predicting recurrent events (AUC 0.71 vs. 0.60). This cohort is a truly external validation cohort for BRUGADA-RISK score but an internal validation cohort for PAT score (25). More external validation is still pending, but its design highlights the importance of considering recurrent arrhythmic burden in risk assessment.

When considered together, the major BrS risk scores demonstrate incremental improvements in predictive performance but also share common limitations. For predicting a first arrhythmic event, the discriminative ability ranged from highest to lowest as follows: BRUGADA-RISK (AUC 0.81, 95% CI 0.71–0.91), PAT (AUC 0.79, 95% CI 0.45–1.12), Delise (AUC 0.77, 95% CI 0.72–0.81), Sieira (AUC 0.73, 95% CI 0.64–0.82), and Shanghai (AUC 0.69, 95% CI 0.61–0.76). For predicting either a first or recurrent event, the PAT score showed the best performance (AUC 0.84, 95% CI 0.59–1.10), followed by Sieira (AUC 0.79, 95% CI 0.74–0.84) and Shanghai (AUC 0.76, 95% CI 0.72–0.81).

Most scores incorporate syncope, ECG parameters (spontaneous type 1 Brugada pattern, early repolarization pattern, fragmented QRS, and T-peak to T-end duration ≥100 ms) that frequently included predictors in the final risk models, and EPS inducibility, though weighting varies. The PAT Score uniquely addresses recurrent arrhythmic events (12), while BRUGADA-RISK applies machine-learning for continuous probabilities (13). Despite these advances, none of these models have been adopted into international guidelines. Current ESC and AHA/ACC recommendations continue to recognize only two factors—arrhythmic syncope and EPS inducibility—as validated predictors sufficient to guide ICD implantation (11, 26). This reflects the methodological fragility of existing scores, the small number of outcome events in most registries, and concerns about reproducibility across diverse populations. Beyond headline discrimination, several methodological aspects differentiate these models. Endpoint definitions also vary: some cohorts include appropriate ICD therapies, others restrict to documented VF or SCD, and follow-up length differs widely. These choices can inflate or deflate apparent performance and complicate cross-model comparisons. Moreover, case-mix and treatment era effects are important—patients treated in the 2000s differ from those enrolled in later years when fever avoidance, drug precautions, and screening improved. Harmonized external validation using the same endpoints and time horizons is needed to fairly benchmark models. Finally, few scores explicitly address competing risks (e.g., non-arrhythmic mortality) or provide dynamic updates over time; pragmatic deployment will require models that can be recalibrated as patient data evolve.

### Key predictors across models

Predictors repeatedly appear across models, though not all are guideline-endorsed. Arrhythmic syncope is a strong predictor, with hazard ratios reported up to the four to five-fold range in several registries (12). Differentiating vasovagal from arrhythmic syncope remains clinically challenging, yet even conservative definitions retain prognostic signal (27). EPS inducibility is more controversial; its predictive value varies depending on stimulation sites, number of extrastimuli, and endpoints. Nevertheless, a pooled analysis demonstrated that inducibility has prognostic utility, contributing to its inclusion in guideline recommendations (12, 28). Spontaneous type 1 ECG is consistently associated with a two- to four-fold increased risk across cohorts (12, 29), although effect sizes differ by ethnicity and age (18, 30). Beyond these core markers, several ECG and clinical features have been proposed. Fragmented QRS reflects depolarization abnormalities and has been associated with events, but definitions differ and interobserver variability is nontrivial (31). Inferolateral early repolarization (ER) patterns have been linked to VF in BrS, but signal-to-noise is modest and the incremental value over baseline type 1 is uncertain (32). Prolonged PR interval suggests broader conduction disease and may identify a subset with more extensive sodium channel involvement (32). Genetic testing frequently identifies *SCN5A* variants, which carry a modestly elevated risk but exhibit incomplete penetrance and variable expressivity (33, 34). Family history of SCD shows an inconsistent association with MAE in BrS; however, when the affected relative died before age 40, the risk appears significantly higher (35). In sum, although multiple factors correlate with outcomes, international guidelines endorse only syncope and EPS as validated predictors sufficient to guide management (11, 26).

### Prediction in true asymptomatic patients with Brugada ECG pattern

Contemporary data have significantly lowered the estimated arrhythmic risk in BrS, an essential consideration when evaluating any risk score. While early registries suggested annual event rates near 1% in asymptomatic individuals, recent large prospective cohorts demonstrate that truly asymptomatic patients experience ventricular arrhythmic events at only 0.2% per year (10). This sharply reduced absolute risk fundamentally limits the performance of current and future risk models, as the rarity of events constrains positive predictive value even when discrimination appears acceptable. These constraints are particularly relevant in the only subgroup in whom risk stratification is clinically meaningful: asymptomatic individuals with a spontaneous type 1 ECG pattern. Symptomatic patients already fall into a high-risk category, whereas those with only drug-induced type 1 ECG have extremely low event rates, leaving asymptomatic spontaneous type 1 patients as the true “grey zone” for ICD decision-making. Yet, real-world comparative data in this specific population remain scarce; to date, only one study—limited to a Japanese cohort—has directly compared leading scores such as PAT and BRUGADA-RISK in asymptomatic patients (25). Thus, while risk scores offer structured decision support, their ability to meaningfully refine ICD eligibility in contemporary practice is inherently constrained.

### Limitations of current models

BrS risk scores face multiple limitations that impede widespread adoption. First, sample sizes are modest and event rates low in most registries, raising concerns about overfitting and limited transportability. Even when cohorts are pooled, heterogeneity in inclusion criteria and follow-up biases discrimination. Second, ethnicity and geography materially affect risk. Third, EPS protocols differ substantially among centers in terms of stimulation sites, coupling intervals, and number of extrastimuli, undermining reproducibility. Fourth, BrS is intrinsically dynamic:fever, drugs, autonomic tone, and circadian variation can unmask or suppress the phenotype, yet current scores rely on static baseline measurements and do not update with time. Fifth, outcome definitions frequently focus on the first arrhythmic event, overlooking recurrent VF and ICD shocks that drive long-term morbidity; the PAT Score is a notable attempt to address this gap. Sixth, most models do not incorporate the competing risks and burdens of ICD therapy itself—such as inappropriate shocks, lead failure, infection, and psychosocial impact—which are central to patient-centered decisions in asymptomatic BrS. Finally, genetics remains difficult to integrate: while SCN5A mutations and rare variants may indicate higher risk, incomplete penetrance and pleiotropy limit their standalone utility, and polygenic architectures are not yet clinically mature.

### Integration of imaging into risk stratification

Advanced imaging, particularly cardiac MRI, has identified right ventricular structural and tissue characteristics suggestive of underlying substrate; integrating such biomarkers may enhance discrimination (36). However, cardiac MRI should not be applied universally in BrS. Global access, cost, and patient tolerability make routine CMR impractical, and current imaging biomarkers require prospective validation before incorporation into formal risk models. A more feasible strategy is selective use of CMR in asymptomatic patients with a spontaneous type 1 ECG whose risk profile remains uncertain after evaluation with established clinical and ECG predictors. In this tiered framework, imaging serves as an adjunctive modifier rather than a primary determinant of management.

### Opportunities for improvement

Across these studies, deep learning applied to 12-lead ECGs and wearable-recorded signals demonstrated strong capability to identify BrS, outperforming traditional clinician-based interpretation. Wearable technology combined with artificial intelligence (AI) further improved detection of dynamic BrS features, suggesting value for continuous, real-world monitoring (37, 38). In SCN5A-positive families, mutation type and polygenic risk scores were variably associated with BrS phenotype, highlighting genetic heterogeneity and the need for integrated genomic-clinical risk assessment (39). Machine learning can identify BrS without invasive drug challenges and often outperforms expert cardiologists in accuracy and diagnostic metrics. By handling complex datasets, AI can classify ambiguous genetic variants and improving disease characterization and enabling more individualized BrS management (40, 41). However, opportunities for advancing BrS risk prediction must balance innovation with clinical responsibility. AI applied to raw ECG signals may uncover subtle substrate features, but current models are non-interpretable and lack prospective validation. As such, AI should function only as a supportive tool; particularly when informing irreversible decisions such as ICD implantation.

## Discussion

In clinical practice, shared decision-making is essential. For asymptomatic BrS, the decision to implant an ICD hinges not only on estimated arrhythmic risk but also on the patient's values, life goals, occupational exposures, and tolerance for device-related complications. Ethical considerations include the implications of genetic testing for family members, uncertainties inherent in probabilistic prediction, and the potential anxiety induced by surveillance. Clinicians should contextualize numerical risk estimates within this broader framework.

Looking ahead, the most promising advances will likely come from combining dynamic physiologic data streams, interpretable AI models, imaging biomarkers, and genomic context, all trained and validated in large, diverse datasets. Such an approach can yield risk scores that are accurate, generalizable, and clinically usable. Until then, the optimal strategy is deliberate, patient-centered care that leverages existing guideline-endorsed predictors while thoughtfully incorporating emerging tools. These priorities should guide research, model development, and clinical practice throughout the next decade and beyond. Cost-effectiveness and patient-reported outcomes should also inform deployment. An ICD that prevents a rare arrhythmic death but generates years of inappropriate shocks may not align with a patient's goals or with health-system value. Decision models that combine calibrated risk estimates with device complication rates, quality-of-life utilities, and patient preferences can surface the trade-offs explicitly and guide truly individualized recommendations. Transparent communication is essential: clinicians should discuss absolute risk, uncertainty intervals, and how preventive behaviors (e.g., fever management, avoidance of culprit drugs) interact with risk projections. Post-implant trajectories also matter: scores could flag patients for closer follow-up after a first ICD therapy or during high-risk physiologic states such as systemic illness. Embedding such logic within electronic health records would facilitate timely, context-aware decision support without adding burden. In parallel, prospective impact studies should measure safety, equity, usability, and cost in routine care settings. across diverse populations worldwide.

## Conclusion

BrS risk stratification has progressed from a binary paradigm to multivariable scores that synthesize clinical, ECG, EPS, and genetic information. Despite this evolution, guidelines remain appropriately conservative, endorsing only syncope and EPS as validated predictors. This caution reflects modest event numbers, heterogeneity across cohorts, and the need for robust external validation. Contemporary models—Sieira, BRUGADA-RISK, PAT, and Shanghai score—provide incremental gains, but none yet demonstrate the breadth of evidence required for formal adoption. A pragmatic path forward is to treat risk scores as decision-support tools that inform, but do not dictate, management.

## References

[1] Brugada P Brugada J. Right bundle branch block, persistent ST segment elevation and sudden cardiac death: a distinct clinical and electrocardiographic syndrome. A multicenter report. J Am Coll Cardiol. (1992) 20(6):1391–6. 10.1016/0735-1097(92)90253-J 1309182

[2] Rattanawong P Mead-Harvey C Fatunde OA Van Der Walt C Ko NK Hooke P Prevalence and incidence of type 1 brugada pattern: a 30-year experience at mayo clinic. Mayo Clin Proc. (2025) 100(1):80–93. 10.1016/j.mayocp.2024.05.028 39641715

[3] Rattanawong P Ngarmukos T Chung EH Vutthikraivit W Putthapiban P Sukhumthammarat W Prevalence of brugada ECG pattern in Thailand from a population-based cohort study. J Am Coll Cardiol. (2017) 69(10):1355–6. 10.1016/j.jacc.2016.12.028 28279299

[4] Vutthikraivit W Rattanawong P Putthapiban P Sukhumthammarat W Vathesatogkit P Ngarmukos T Worldwide prevalence of brugada syndrome: a systematic review and meta-analysis. Acta Cardiol Sin. (2018) 34(3):267–77. 10.6515/ACS.201805_34(3).20180302B 29844648 PMC5968343

[5] Vatta M Dumaine R Varghese G Richard TA Shimizu W Aihara N Genetic and biophysical basis of sudden unexplained nocturnal death syndrome (SUNDS), a disease allelic to brugada syndrome. Hum Mol Genet. (2002) 11(3):337–45. 10.1093/hmg/11.3.337 11823453

[6] Gehi AK Duong TD Metz LD Gomes JA Mehta D. Risk stratification of individuals with the brugada electrocardiogram: a meta-analysis. J Cardiovasc Electrophysiol. (2006) 17(6):577–83. 10.1111/j.1540-8167.2006.00455.x 16836701

[7] Priori SG Blomstrom-Lundqvist C Mazzanti A Blom N Borggrefe M Camm J 2015 ESC guidelines for the management of patients with ventricular arrhythmias and the prevention of sudden cardiac death: the task force for the management of patients with ventricular arrhythmias and the prevention of sudden cardiac death of the European Society of Cardiology (ESC)Endorsed by: association for European paediatric and congenital cardiology (AEPC). Europace. (2015) 17(11):1601–87. 10.1093/europace/euv319 26318695

[8] Wilde AAM Saenen J. Risk stratification in brugada syndrome: how low can we go? Circulation. (2023) 148(20):1556–8. 10.1161/CIRCULATIONAHA.123.066697 37956226

[9] Delise P. Risk stratification in brugada syndrome: the challenge of the grey zone. Eur Heart J. (2021) 42(17):1696–7. 10.1093/eurheartj/ehaa1100 33428713

[10] Gaita F Cerrato N Giustetto C Martino A Bergamasco L Millesimo M Asymptomatic patients with brugada ECG pattern: long-term prognosis from a large prospective study. Circulation. (2023) 148(20):1543–55. 10.1161/CIRCULATIONAHA.123.064689 37830188 PMC10637308

[11] Zeppenfeld K Tfelt-Hansen J de Riva M Winkel BG Behr ER Blom NA 2022 ESC guidelines for the management of patients with ventricular arrhythmias and the prevention of sudden cardiac death. Eur Heart J. (2022) 43(40):3997–4126. 10.1093/eurheartj/ehac262 36017572

[12] Rattanawong P Mattanapojanat N Mead-Harvey C Van Der Walt C Kewcharoen J Kanitsoraphan C Predicting arrhythmic event score in brugada syndrome: worldwide pooled analysis with internal and external validation. Heart Rhythm. (2023) 20(10):1358–67. 10.1016/j.hrthm.2023.06.013 37355026

[13] Honarbakhsh S Providencia R Garcia-Hernandez J Martin CA Hunter RJ Lim WY A primary prevention clinical risk score model for patients with brugada syndrome (BRUGADA-RISK). JACC Clin Electrophysiol. (2021) 7(2):210–22. 10.1016/j.jacep.2020.08.032 33602402

[14] Sieira J Conte G Ciconte G Chierchia GB Casado-Arroyo R Baltogiannis G A score model to predict risk of events in patients with brugada syndrome. Eur Heart J. (2017) 38(22):1756–63. 10.1093/eurheartj/ehx119 28379344

[15] Antzelevitch C Yan GX Ackerman MJ Borggrefe M Corrado D Guo J J-Wave syndromes expert consensus conference report: emerging concepts and gaps in knowledge. Heart Rhythm. (2016) 13(10):e295–324. 10.1016/j.hrthm.2016.05.024 27423412 PMC5035208

[16] Priori SG Gasparini M Napolitano C Della Bella P Ottonelli AG Sassone B Risk stratification in brugada syndrome: results of the PRELUDE (PRogrammed ELectrical stimUlation preDictive valuE) registry. J Am Coll Cardiol. (2012) 59(1):37–45. 10.1016/j.jacc.2011.08.064 22192666

[17] Probst V Veltmann C Eckardt L Meregalli PG Gaita F Tan HL Long-term prognosis of patients diagnosed with brugada syndrome: results from the FINGER brugada syndrome registry. Circulation. (2010) 121(5):635–43. 10.1161/CIRCULATIONAHA.109.887026 20100972

[18] Milman A Andorin A Postema PG Gourraud JB Sacher F Mabo P Ethnic differences in patients with brugada syndrome and arrhythmic events: new insights from survey on arrhythmic events in brugada syndrome. Heart Rhythm. (2019) 16(10):1468–74. 10.1016/j.hrthm.2019.07.003 31284050

[19] Milman A Andorin A Gourraud JB Sacher F Mabo P Kim SH Age of first arrhythmic event in brugada syndrome: data from the SABRUS (survey on arrhythmic events in brugada syndrome) in 678 patients. Circ Arrhythm Electrophysiol. (2017) 10(12):1–15. 10.1161/CIRCEP.117.005222 29254945

[20] Delise P Allocca G Marras E Giustetto C Gaita F Sciarra L Risk stratification in individuals with the brugada type 1 ECG pattern without previous cardiac arrest: usefulness of a combined clinical and electrophysiologic approach. Eur Heart J. (2011) 32(2):169–76. 10.1093/eurheartj/ehq381 20978016 PMC3021386

[21] Kawazoe H Nakano Y Ochi H Takagi M Hayashi Y Uchimura Y Risk stratification of ventricular fibrillation in brugada syndrome using noninvasive scoring methods. Heart Rhythm. (2016) 13(10):1947–54. 10.1016/j.hrthm.2016.07.009 27424075

[22] Okamura H Kamakura T Morita H Tokioka K Nakajima I Wada M Risk stratification in patients with brugada syndrome without previous cardiac arrest - prognostic value of combined risk factors. Circ J. (2015) 79(2):310–7. 10.1253/circj.CJ-14-1059 25428522

[23] Gomes DA Lambiase PD Schilling RJ Cappato R Adragao P Providencia R. Multiparametric models for predicting major arrhythmic events in brugada syndrome: a systematic review and critical appraisal. Europace. (2025) 27(5):1–13. 10.1093/europace/euaf091 PMC12092914 40314213

[24] Wei HT Liu W Ma YR Chen S. Performance of multiparametric models in patients with brugada syndrome: a systematic review and meta-analysis. Front Cardiovasc Med. (2022) 9:859771. 10.3389/fcvm.2022.859771 35497979 PMC9047913

[25] Kamakura T Takagi M Komatsu Y Shinohara T Aizawa Y Sekiguchi Y Validation of novel risk prediction models in patients with brugada syndrome: a multicenter study in Japan. Heart Rhythm. (2025) 22(7):1710–7. 10.1016/j.hrthm.2024.09.024 39288881

[26] Al-Khatib SM Stevenson WG Ackerman MJ Bryant WJ Callans DJ Curtis AB 2017 AHA/ACC/HRS guideline for management of patients with ventricular arrhythmias and the prevention of sudden cardiac death: executive summary: a report of the American College of Cardiology/American Heart Association task force on clinical practice guidelines and the heart rhythm society. Heart Rhythm. (2018) 15(10):e190–252. 10.1016/j.hrthm.2017.10.035 29097320

[27] Rattanawong P Kewcharoen J Yinadsawaphan T Fatunde OA Kanitsoraphan C Vutthikraivit W Type of syncope and outcome in brugada syndrome: a systematic review and meta-analysis. J Arrhythm. (2023) 39(2):111–20. 10.1002/joa3.12822 37021016 PMC10068940

[28] Sroubek J Probst V Mazzanti A Delise P Hevia JC Ohkubo K Programmed ventricular stimulation for risk stratification in the brugada syndrome: a pooled analysis. Circulation. (2016) 133(7):622–30. 10.1161/CIRCULATIONAHA.115.017885 26797467 PMC4758872

[29] Letsas KP Liu T Shao Q Korantzopoulos P Giannopoulos G Vlachos K Meta-Analysis on risk stratification of asymptomatic individuals with the brugada phenotype. Am J Cardiol. (2015) 116(1):98–103. 10.1016/j.amjcard.2015.03.044 25933735

[30] Minier M Probst V Berthome P Tixier R Briand J Geoffroy O Age at diagnosis of brugada syndrome: influence on clinical characteristics and risk of arrhythmia. Heart Rhythm. (2020) 17(5 Pt A):743–9. 10.1016/j.hrthm.2019.11.027 31790831

[31] Morita H Kusano KF Miura D Nagase S Nakamura K Morita ST Fragmented QRS as a marker of conduction abnormality and a predictor of prognosis of brugada syndrome. Circulation. (2008) 118(17):1697–704. 10.1161/CIRCULATIONAHA.108.770917 18838563

[32] Vitali F Brieda A Balla C Pavasini R Tonet E Serenelli M Standard ECG in brugada syndrome as a marker of prognosis: from risk stratification to pathophysiological insights. J Am Heart Assoc. (2021) 10(10):e020767. 10.1161/JAHA.121.020767 33977759 PMC8200706

[33] Yamagata K Horie M Aiba T Ogawa S Aizawa Y Ohe T Genotype-Phenotype correlation of SCN5A mutation for the clinical and electrocardiographic characteristics of probands with brugada syndrome: a Japanese multicenter registry. Circulation. (2017) 135(23):2255–70. 10.1161/CIRCULATIONAHA.117.027983 28341781

[34] Hosseini SM Kim R Udupa S Costain G Jobling R Liston E Reappraisal of reported genes for sudden arrhythmic death: evidence-based evaluation of gene validity for brugada syndrome. Circulation. (2018) 138(12):1195–205. 10.1161/CIRCULATIONAHA.118.035070 29959160 PMC6147087

[35] Rattanawong P Kewcharoen J Kanitsoraphan C Barry T Shanbhag A Ko Ko NL Does the age of sudden cardiac death in family members matter in brugada syndrome? J Am Heart Assoc. (2021) 10(11):e019788. 10.1161/JAHA.120.019788 34013737 PMC8483509

[36] Pappone C Santinelli V Mecarocci V Tondi L Ciconte G Manguso F Brugada syndrome: new insights from cardiac magnetic resonance and electroanatomical imaging. Circ Arrhythm Electrophysiol. (2021) 14(11):e010004. 10.1161/CIRCEP.121.010004 34693720

[37] Liu CM Liu CL Hu KW Tseng VS Chang SL Lin YJ A deep learning-enabled electrocardiogram model for the identification of a rare inherited arrhythmia: brugada syndrome. Can J Cardiol. (2022) 38(2):152–9. 10.1016/j.cjca.2021.08.014 34461230

[38] Liao S Bokhari M Chakraborty P Suszko A Jones G Spears D Use of wearable technology and deep learning to improve the diagnosis of brugada syndrome. JACC Clin Electrophysiol. (2022) 8(8):1010–20. 10.1016/j.jacep.2022.05.003 35981788

[39] Wijeyeratne YD Tanck MW Mizusawa Y Batchvarov V Barc J Crotti L SCN5A Mutation type and a genetic risk score associate variably with brugada syndrome phenotype in SCN5A families. Circ Genom Precis Med. (2020) 13(6):e002911. 10.1161/CIRCGEN.120.002911 33164571 PMC7748043

[40] Sahebnasagh M Farjoo MH. Artificial intelligence for brugada syndrome diagnosis and gene variants interpretation. Am J Cardiovasc Dis. (2025) 15(1):1–12. 10.62347/YQHQ1079 40124093 PMC11928888

[41] Calburean PA Pannone L Monaco C Rocca DD Sorgente A Almorad A Predicting and recognizing drug-induced type I brugada pattern using ECG-based deep learning. J Am Heart Assoc. (2024) 13(10):e033148. 10.1161/JAHA.123.033148 38726893 PMC11179812

